# Apoptosis, Toll-like, RIG-I-like and NOD-like Receptors Are Pathways Jointly Induced by Diverse Respiratory Bacterial and Viral Pathogens

**DOI:** 10.3389/fmicb.2017.00276

**Published:** 2017-03-01

**Authors:** Isidoro Martínez, Juan C. Oliveros, Isabel Cuesta, Jorge de la Barrera, Vicente Ausina, Cristina Casals, Alba de Lorenzo, Ernesto García, Belén García-Fojeda, Junkal Garmendia, Mar González-Nicolau, Alicia Lacoma, Margarita Menéndez, David Moranta, Amelia Nieto, Juan Ortín, Alicia Pérez-González, Cristina Prat, Elisa Ramos-Sevillano, Verónica Regueiro, Ariel Rodriguez-Frandsen, Dolores Solís, José Yuste, José A. Bengoechea, José A. Melero

**Affiliations:** ^1^Centro Nacional de Microbiología, Instituto de Salud Carlos IIIMadrid, Spain; ^2^Centro de Investigación Biomédica en Red de Enfermedades Respiratorias (CIBERES), Instituto de Salud Carlos IIIMadrid, Spain; ^3^Centro Nacional de Biotecnología (CSIC)Madrid, Spain; ^4^Servei de Microbiologia, Hospital Universitari Germans Trias i Pujol, Badalona, Institut d' Investigació Germans Trias i Pujol, Universitat Autònoma de BarcelonaBarcelona, Spain; ^5^Departmento de Bioquímica y Biología Molecular I, Universidad ComplutenseMadrid, Spain; ^6^Centro de Investigaciones Biológicas (CSIC)Madrid, Spain; ^7^Instituto de Agrobiotecnología, CSIC-Universidad Pública de Navarra-GobNavarra, Spain; ^8^Fundación de Investigación Sanitaria de las Islas Baleares, Instituto de Investigación Sanitaria de PalmaPalma, Spain; ^9^Instituto de Química Física Rocasolano (CSIC)Madrid, Spain

**Keywords:** respiratory pathogens, host response, core of up-regulated genes, bacterial infections, viral infections

## Abstract

Lower respiratory tract infections are among the top five leading causes of human death. Fighting these infections is therefore a world health priority. Searching for induced alterations in host gene expression shared by several relevant respiratory pathogens represents an alternative to identify new targets for wide-range host-oriented therapeutics. With this aim, alveolar macrophages were independently infected with three unrelated bacterial (*Streptococcus pneumoniae, Klebsiella pneumoniae*, and *Staphylococcus aureus*) and two dissimilar viral (respiratory syncytial virus and influenza A virus) respiratory pathogens, all of them highly relevant for human health. Cells were also activated with bacterial lipopolysaccharide (LPS) as a prototypical pathogen-associated molecular pattern. Patterns of differentially expressed cellular genes shared by the indicated pathogens were searched by microarray analysis. Most of the commonly up-regulated host genes were related to the innate immune response and/or apoptosis, with Toll-like, RIG-I-like and NOD-like receptors among the top 10 signaling pathways with over-expressed genes. These results identify new potential broad-spectrum targets to fight the important human infections caused by the bacteria and viruses studied here.

## Introduction

According to the World Health Organization, lower respiratory tract infections are the fourth leading cause of human death worldwide (3.1 million deaths in 2012), only behind ischemic heart disease, stroke and chronic obstructive pulmonary disease (COPD) (http://www.who.int/mediacentre/factsheets/fs310/en/). Acute respiratory infections are the most frequent cause of visits to health services around the world, and account for 20–40% of children hospitalizations, with pneumonia being the leading global killer of children under five (UNICEF and WHO, 2006^1^; Forum of International Respiratory Societies, 2013^2^) Consequently, respiratory infections constitute an enormous global health burden, complicated by the existence of different etiologic agents that frequently co-infect the same individual, the emergence of drug-resistant variants, and the emergence and re-emergence of new human respiratory pathogens.

Infectious diseases can be regarded as the result of specific interactions between pathogens and their hosts. Classical approaches to combat these infections have focused on targeting distinctive processes of each pathogen's infectious cycle with specific drugs. The rationale behind this approach is that the more specific the target, the less likely the drug will cause host toxicity. Despite the unquestionable success of this approach in different areas, the high economic cost of developing multiple pathogen-specific treatments and the emergence of drug resistance have compelled searching for alternative therapeutic approaches.

Promising alternative strategies to overcome the noted problems of pathogen specificity and drug resistance might arise from studies of the host response to invading pathogens. Modulation of host genes essential for the infectivity of multiple microorganisms is conceptually possible since distinct pathogens confront the same host background and may converge onto similar cellular responses (Jenner and Young, 2005). The discovery of host gene products that might be targets of effective drugs against multiple infectious agents, i.e., host-oriented broad-spectrum (HOBS) drug targets, would have several benefits with respect to treating multiple, co-infecting microorganisms, lowering economic costs and limiting drug resistance selection. An essential first step in the discovery of such drug targets is to identify processes and cellular pathways altered in common by multiple pathogens.

The identification of biological pathways induced by different viruses or bacteria has been the topic of recent work (Zinman et al., 2011; Smith et al., 2012, 2013; Kidane et al., 2013). Most of these studies, however, examined separately viruses and bacteria, and were mostly based on large-scale analysis of previously published microarray datasets (Smith et al., 2012, 2013; Kidane et al., 2013). In only one report, alveolar macrophages from mice and macaque were infected in parallel with influenza A virus (IAV), *Mycobacterium tuberculosis* and *Francisella tularensis* and the innate immune responses were compared (Zinman et al., 2011). A core of activated genes was identified in both animal species and across the three pathogens. However, both IAV and *M. tuberculosis* have mechanisms aimed to minimize the induction of innate immunity, thus partially hiding the full-blown of the host response (Geiss et al., 2002; Nau et al., 2002). Therefore, data from additional microorganisms are clearly needed to build a solid picture of a possible common response against respiratory pathogens.

The aim of this study was to search for collective mechanisms triggered by different respiratory pathogens when infecting alveolar macrophages, one of the first cell types of the innate immune system to interact with pathogens invading the lung. Thus, the macrophage-derived cell line MH-S was infected independently with five unrelated respiratory pathogens, highly relevant for humans; i.e., two human viruses (IAV, and respiratory syncytial virus, RSV) and three bacteria (*Klebsiella pneumoniae, Streptococcus pneumoniae*, and *Staphylococcus aureus*).

IAV is considered a major, global health threat that causes yearly winter worldwide epidemics in humans, with 50 million annual illnesses in the United States (Peasah et al., 2013). Symptoms include fever, headache, cough, sore throat, nasal congestion, sneezing and body aches. While IAV infects individuals of all age groups, human RSV is the main cause of lower respiratory tract infections (bronchiolitis and pneumonia) in infants and young children, causing annually about 34 million new episodes of acute respiratory infections (ARI) worldwide in children younger than 5 years, with 10% of them requiring hospital admission (Nair et al., 2010). In addition, RSV is also a relevant pathogen among elderly and immunocompromised adults (Falsey, 2007).

*S. pneumoniae* colonizes asymptomatically the human nasopharynx in a high percentage of the population, being the most common causative agent of acute otitis media and community-acquired pneumonia and a major cause of bacterial sepsis and meningitis (O'Brien et al., 2009). *S. aureus* is also a commensal of the human nasopharynx and skin that causes invasive infections particularly worrisome when caused by methicillin-resistant (MRSA) strains which are associated with therapeutic failure and elevated mortality (Mulcahy and McLoughlin, 2016). Together with MRSA, of particular concern is the mounting prevalence of infections caused by multidrug resistant Gram-negative bacteria, *K. pneumoniae* being a very significant challenge (Bengoechea, 2016). *K. pneumoniae* causes a wide range of infections, including pneumonias, urinary tract infections, bacteremias and liver abscesses. Historically, *K. pneumoniae* has caused serious infections primarily in immunocompromised individuals, but the recent emergence and spread of hypervirulent strains have broadened the number of people susceptible to infections to include healthy and immunosufficient individuals (Paczosa and Mecsas, 2016).

In order to identify possible targets for the development of host-oriented broad-spectrum therapies, the differential expression of cellular genes upon exposure of MH-S cells to the above selected pathogens was examined by microarray technology. Shared altered pathways related to apoptosis and pathogen-specific pattern recognition receptors (PRRs) were identified, indicating that macrophages respond to invading bacteria and viruses using similar ammunitions and strategies.

## Materials and methods

### Macrophage culture

MH-S cells (ATCC® CRL-2019™) were originally obtained from bronchoalveolar lavage of BALB/cj mice and were *in vitro* transformed with SV40 (Mbawuike and Herscowitz, 1989). They lack contact inhibition of growth and are trypsin-sensitive, but they maintain typical macrophage morphology, and express cell surface Ia and Mac-1 antigens. MH-S cells were maintained in RPMI 1640 medium supplemented with 10% fetal bovine serum (FBS), 4 mM L-glutamine, 100 U/ml penicillin and 100 U/ml streptomycin. All cells were incubated at 37°C in a 5% CO_2_ atmosphere.

### Virus and bacteria strains

Table 1 shows the viruses and bacteria used to infect MH-S cells.

**Table 1:** **List of respiratory pathogens analyzed in this study**.

**Pathogen**	**Strain**	**Post-infection time (h)**
Respiratory syncytial virus	A2, Long	1, 18
Influenza virus^*^	A/PR/8/34, ΔNS1	7
*Streptococcus pneumoniae*	D39 (NCTC 7466)	1, 4
*Klebsiella pneumoniae*	121 (52145), 995 (43816)	1, 4, 8
*Staphylococcus aureus*	Newman	1, 4

* *Influenza A/PR/8/34 virus and the ΔNS1 mutant were analyzed as an outgroup of the current study for reasons explained in the text*.

The Long and A2 strains of human RSV were propagated in HEp-2 cells and purified from clarified culture supernatants by polyethylene glycol precipitation and centrifugation in a discontinuous sucrose gradient, as previously described (Mbiguino and Menezes, 1991; Martínez et al., 2007).

Influenza wild type A/PR/8/34 and A/PR/8/34 lacking NS1 (A/PR/8/34 ΔNS1) (a gift of A. García-Sastre) viruses were propagated in MDCK and Vero cells, respectively. Wild type virus was titrated by plaque assay in MDCK cells and the ΔNS1 mutant was titrated in MDCK-V2 cells that express the V protein of SV5 which leads to STAT1 degradation (Andrejeva et al., 2002).

*S. pneumoniae* D39 (NCTC 7466) is a serotype 2 strain previously used for many studies dealing with pneumococcal pathogenesis (Lanie et al., 2007). *S. pneumoniae* cells were grown in C medium supplemented with 0.08% yeast extract at 37°C as previously reported (Domenech et al., 2009). *S. aureus* (Newman strain) and *K. pneumoniae* (52145 and 43186 strains) were grown in 5 ml of Luria Bertani medium (180 rpm, 37°C) and harvested in the exponential phase.

### MH-S infections and exposure to LPS

MH-S subconfluent monolayers were either mock-infected or infected with RSV at a multiplicity of infection (MOI) of 3 plaque forming units (pfu) per cell. Cells were incubated with the viral inoculum in RPMI 1640 supplemented with 2% FBS (RPMI2) for 90 min at 37°C. After that time, the inoculum was removed and fresh RPMI2 was added. At 1 and 18 h post-infection, culture supernatants were removed, cells were trypsinized, washed with PBS and RNA was extracted. Infection of MH-S with influenza virus was done similarly at a MOI of 3 pfu/cell in RPMI 1640 and harvested 7 h after infection.

For *S. aureus, S. pneumoniae* and *K. pneumoniae* infections, MH-S cells were incubated with bacteria at a MOI of 50 bacteria/cell for 30 min. Subsequently, cells were washed with PBS; then, fresh medium with gentamicin (100 μg/ml) and penicillin (100 U/ml) was added to kill extracellular bacteria and prevent further reinfections. At 1, 4, or 8 h post-infection (Table 1), cells were harvested, and total RNA extracted and stored at −80°C until analysis. In addition, MH-S subconfluent monolayers were also incubated in quadruplicate with 1 μg/ml of smooth LPS from *Escherichia coli* O55B5 (Sigma-Aldrich, St. Louis, MO, U.S.A.) or with buffer for 1 h. Then, culture supernatants were removed and cells were scraped and washed with PBS before RNA extraction.

### RNA isolation

Total RNA from mock-infected or infected cells was purified with the RNeasy Minikit (Qiagen) following the manufacturer's instruction. Briefly, cells were lysed in the presence of guanidine-thiocyanate containing buffer to inactivate RNases and ethanol was added (70% final) to facilitate binding of mRNAs to RNeasy spin column silica membranes. After washing, RNAs were eluted from the membranes in a small volume of RNase-free water.

### Gene expression profiling: sample labeling and microarray hybridizations

Gene expression profiling analyses were conducted using the Agilent Whole Mouse Genome microarray (G4122F, Agilent Technologies, Palo Alto, CA), representing about 44,000 mouse genes, transcripts and controls.

One microgram of total RNA from each sample was amino-allyl labeled and amplified using the Amino Allyl MessageAmp II aRNA Amplification Kit (Applied Biosystems, Foster City, CA). Aliquots (1.2 μg) of antisense amplified RNA were fluorescently labeled using Cy3, according to manufacturer's instructions, and then appropriately hybridized to Agilent oligomicroarrays. Each sample was directly hybridized on a single microarray. After washing, microarray slides were scanned using a Gene Pix 4100A scanner (Molecular Devices, Inc).

### Microarray data transformation and normalization

Extraction of numerical data from scanned microarray images (one channel per slide) was carried out using the GenePix Pro software (Molecular Devices, Inc). Median intensity values for each spot were used in subsequent analysis. Background signal was removed by the normexp method (Silver et al., 2009) with an offset of 50. Background-removed intensities were normalized by quantile adjustment of all replicates involved in each comparison. Both background removal and data normalization steps were performed with limma package (Ritchie et al., 2015) (functions *normexp* and *normalizeQuantiles*, respectively).

### Data analysis

For each comparison (infected sample *versus* non infected sample) with their replicates, differential expression (log Ratios) and statistical significance values (*p* values) were calculated by limma (Ritchie et al., 2015) with default parameters for one-channel microarray data. Log Ratios were transformed to linear Fold Changes and *p* values were adjusted by FDR (Benjamini and Hochberg, 1995) to minimize the multiple testing problem.

### Functional annotation of candidate genes

Functional annotation of selected genes was carried out using DAVID (Database for Annotation, Visualization and Integrated Discovery) Bioinformatics Resources 6–7 (Huang et al., 2009a,b). Kyoto Encyclopedia of Genes and Genomes (KEGG) pathways figures were obtained with KEGG Mapper tool available at KEGG database (Kanehisa and Goto, 2000; Du et al., 2014). Colors of selected genes are based on log Ratios.

### Microarray data availability

Raw and normalized data were deposited on GEO-NCBI database with accession id GSE88825

## Results and discussion

### A core of cellular genes is up-regulated in alveolar macrophages by bacterial and viral respiratory pathogens

In this report we explored the possibility of finding host genes collectively de-regulated by various respiratory pathogens. To this end, cells of the alveolar macrophage murine cell line MH-S were independently infected with RSV, IAV, *K. pneumoniae, S. pneumoniae*, or *S. aureus* (Table 1), followed by microarray analysis of cellular gene expression at different times after infection. In some cases, more than one strain (RSV and *K. pneumoniae*) was included in the study to verify inter-strain reproducibility. Finally, cells were also exposed to lipopolysaccharide (LPS) as an archetypal Gram-negative bacterial agonist.

It has been reported that the NS1 protein of IAV is a potent inhibitor of the cellular antiviral response (Geiss et al., 2002). Therefore, MH-S cells infected with either the wild type influenza virus A/PR/8/34 strain or with a mutant lacking the NS1 gene were analyzed as an outgroup and then compared with the results from the other pathogens.

**Figure 1:**
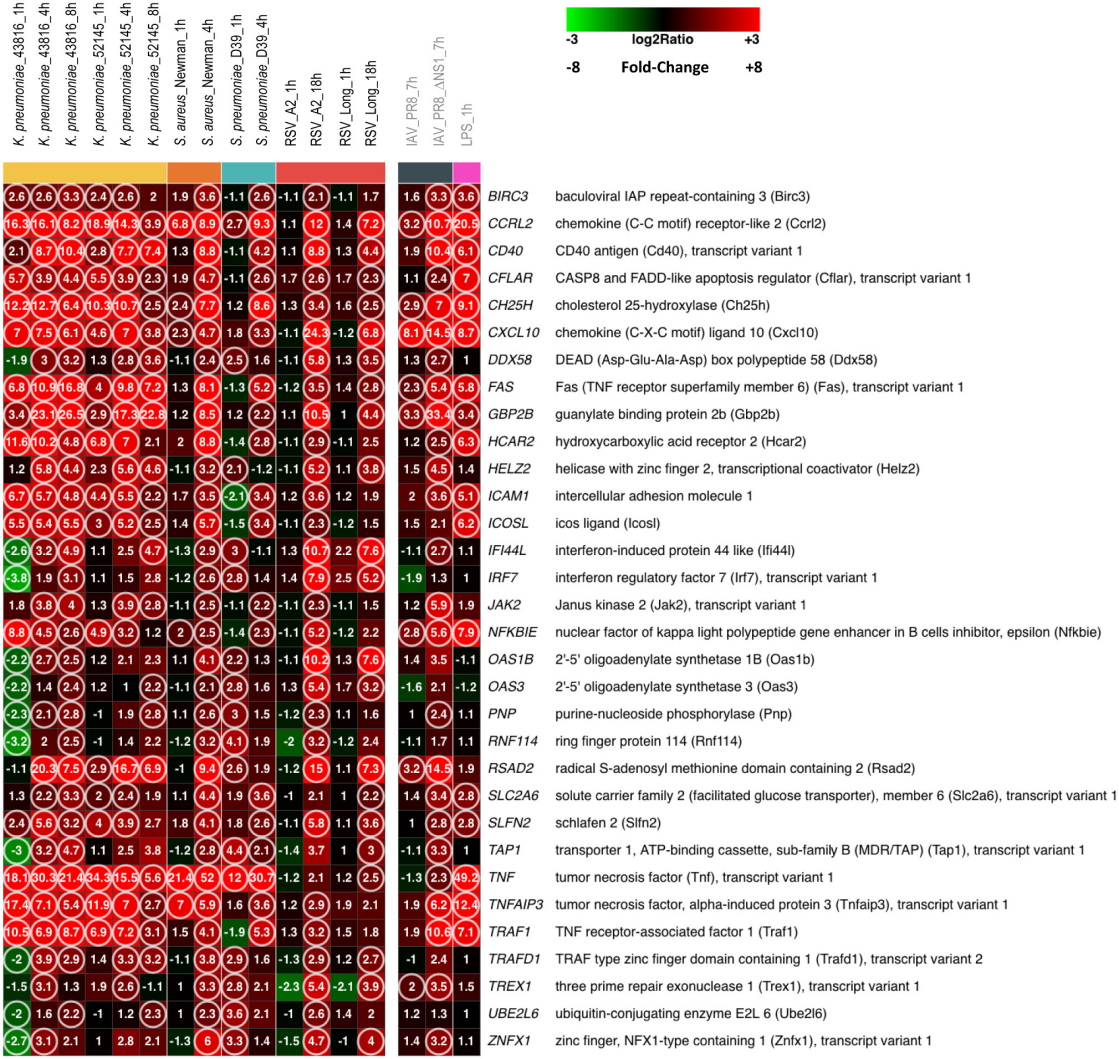
**A core of host genes up-regulated by different respiratory bacteria and viruses**. MH-S cells were infected with the bacteria and viruses indicated at the top of each lane, for the noted periods of time. In addition, MH-S cells were also incubated with LPS for 1 hour. At the end of the incubation period, RNA was extracted, amplified, labeled and used to hybridize Agilent Whole Mouse Genome microarrays that were analyzed as indicated in Materials and Methods. The Figure shows the heatmap of 32 genes commonly up-regulated, considering any time point after infection by the indicated bacteria or RSV, using the following statistical parameters: Fold Change (FC) ≥ 2 compared with mock infected controls and False Discovery Rate (FDR) ≤ 0.1. Genes are identified and displayed in alphabetical order. The IAV and LPS results are shown detached at right since they were not used for the identification of the 32 genes listed in the Figure. Numbers refer to FC values for each noted gene of each pathogen at the post-infection time indicated at the top. The circles indicate genes with FC ≥ 2 and FDR ≤ 0.1. Squares are colored using the scale shown at the top of the Figure.

Figure 1 shows the heatmap of 32 genes that were found commonly up-regulated (fold change, FC ≥ 2.0 and false discovery rate, FDR ≤ 0.1) at any given time after infection with *K. pneumoniae, S. aureus, S. pneumoniae*, or RSV in comparison to mock-infected MH-S cells as a control (the list of genes is shown in detail in Supplementary File S1). No commonly down-regulated genes were found under these conditions (FC ≥ 2.0, FDR ≤ 0.1).

Since the infectious cycle of different bacteria differs from each other, two to three different post-infection time points were selected, based on previous experience, for extraction of cellular RNAs and subsequent microarray analysis. Enhanced expression of certain genes was observed just 1 h after infection but the level of up-regulation and the number of commonly up-regulated genes increased substantially after 4 h of bacterial infection (Figure 1). It is worth noting that not only the number of up-regulated genes but also similarities in the patterns and extent of certain changes among the three unrelated bacteria were particularly striking. For instance, tumor necrosis factor (TNF), chemokine receptor-like 2 (Ccrl2) and cholesterol 25-hydroxylase (Ch25h) were, in that order, the three genes with the highest level of enhanced expression. At the other extreme 2′-5′ oligoadenylate synthetase 3 (Oas3), purine-nucleoside phosphorylase (Pnp), and ubiquitin-conjugating enzyme E2L6 (Ube2l6) were among the genes whose expression increased just above the cut-off level of FC ≥ 2.0. Interestingly, changes in gene expression observed after exposure of MH-S cells to LPS resembled to great extent the changes induced by the whole bacteria (Figure 1). Thus, changes in the genes with the highest and lowest level of up-regulation in bacterial-infected cells were reproduced in the LPS-treated cultures. This finding confirms that LPSs can induce the majority of the macrophage activation program, as similarly observed for lipoteichoic acids and the muramyl dipeptide MurNAc-L-Ala-D-isoGlx (Nau et al., 2002).

Previous studies indicated that alterations in gene expression in epithelial cells infected with RSV were observed only after extended periods of time (Martínez et al., 2007). Thus, MH-S cells were infected with two different strains (A2 and Long) of human RSV before harvesting for RNA extraction at 1 and 18 h post-infection. In agreement with previous findings, almost none of the genes shown in Figure 1 was up-regulated 1 h after infection of MH-S cells with RSV. However, expression of the 32 genes shown in Figure 1 was increased, at least for one of the RSV strains, above FC ≥ 2.0 level, 18 h after infection. It is worth noting that the changes observed with the two strains were very similar, adding weight to the results of the analysis.

The number of commonly up-regulated genes dropped to eight when RNA obtained from MH-S cells infected for 7 h with the influenza virus A/PR/8/34 was included in the analysis (Figure 1). However, this number increased to 25 when the NS1 deletion mutant was examined (Figure 1), indicating that ablation of the IAV gene which counteracts the host innate response uncovered a pattern of gene expression alterations that resembles to great extent those seen in macrophages infected by other respiratory pathogens.

When the core of 32 up-regulated genes was sorted into gene ontology categories, the immune response and the cell death/apoptosis categories emerged as the biological processes with the most significant changes in gene expression (Table 2). Accordingly, RIG-I-like, Toll-like and NOD-like receptor signaling pathways as well as apoptosis were among the top 10 pathways to which up-regulated genes could be assigned (Table 3).

**Table 2:** **List of the top ten Gene Ontology Biological Processes over-represented in the genes that are commonly up-regulated by the respiratory pathogens studied (FC ≥ 2, FDR ≤ 0.1)**.

**Term**	**Gene symbol**	**Benjamini (*P*-value)**
GO:0006955 Immune response	DDX58, ICAM1, ICOSL, TNF, IRF7, TAP1, OAS1B, OAS3, RSAD2, FAS, CXCL10	2.4E-06
GO:0002252 Immune effector process	ICAM1, ICOSL, IRF7, RSAD2, FAS	2.0E-02
GO:0012501 Programmed cell death	TRAF1, CFLAR, TNF, JAK2, FAS, TNFAIP3, BIRC3	2.2E-02
GO:0002449 Lymphocyte mediated immunity	ICAM1, ICOSL, IRF7, FAS	2.3E-02
GO:0009615 Response to virus	DDX58, IRF7, OAS1B, RSAD2	2.3E-02
GO:0016265 Death	TRAF1, CFLAR, TNF, JAK2, FAS, TNFAIP3, BIRC3	2.5E-02
GO:0042127 Regulation of cell proliferation	ICOSL, TNF, JAK2, CD40, PNP, SLFN2, CXCL10	2.6E-02
GO:0008219 Cell death	TRAF1, CFLAR, TNF, JAK2, FAS, TNFAIP3, BIRC3	2.6E-02
GO:0002443 Leukocyte mediated immunity	ICAM1, ICOSL, IRF7, FAS	2.6E-02
GO:0006915 Apoptosis	TRAF1, CFLAR, TNF, JAK2, FAS, TNFAIP3, BIRC3	2.7E-02

**Table 3:** **KEGG pathways over-represented in the genes that are commonly up-regulated by the panel of respiratory pathogens studied (FC ≥ 2, FDR ≤ 0.1)**.

**Term**	**Gene symbol**	**Benjamini (*P*-value)**
mmu04622 RIG-I-like receptor signaling pathway	DDX58, TNF, IRF7, CXCL10	2.5E-02
mmu04623 Cytosolic DNA-sensing pathway	DDX58, IRF7, TREX1, CXCL10	2.7E-02
mmu04210 Apoptosis	CFLAR, TNF, FAS, BIRC3	3.4E-02
mmu04620 Toll-like receptor signaling pathway	TNF, IRF7, CD40, CXCL10	3.7E-02
mmu04920 Adipocytokine signaling pathway	TNF, NFKBIE, JAK2	1.1E-01
mmu04621 NOD-like receptor signaling pathway	TNF, TNFAIP3, BIRC3	1.1E-01
mmu05330 Allograft rejection	TNF, FAS, CD40	1.2E-01
mmu04060 Cytokine-cytokine receptor interaction	TNF, FAS, CD40, CXCL10	2.0E-01
mmu04650 Natural killer cell mediated cytotoxicity	ICAM1, TNF, FAS	2.5E-01
mmu04514 Cell adhesion molecules (CAMs)	ICAM1, ICOSL, CD40	3.2E-01
mmu05310 Asthma	TNF, CD40	3.5E-01

Due to the number of pathogens and conditions analyzed in this study, the probability that a specific gene up-regulated by all microorganisms could be a false positive assignment was considered to be very low. However, substantial quantitative differences between different pathogens could be expected at the level of FC induction. This variability might increase FDR values above the ≤ 0.1 threshold and thus decrease the number of genes considered to be up-regulated. Therefore, in order to enlarge the list of up-regulated genes, two additional statistical thresholds, excluding FDR, were used to analyze the microarray data: FC ≥ 2.0 (Supplementary File S2) and FC ≥ 1.5 (Supplementary File S3). As in Figure 1, the genes in the supplementary files are listed in alphabetical order. The next sections hence analyze in detail relevant aspects of the biological processes (Table 2) and biological pathways (Table 3) that include most of the commonly up-regulated genes of Figure 1, but also extended to genes included in Supplementary Files S2, S3.

### Induction and control of the immune response

Most of the genes up-regulated by all pathogens under study were found to be part of Gene Ontology Biological Processes related with the innate immune response (Table 2). This response is necessary to limit pathogen infections, but at the same time it needs to be carefully regulated to avoid immune-mediated host damage. Accordingly, several genes that mediate activation of the immune response were induced by all pathogens, including transcription factors (e.g., IRF7, Figure 1; IRF1, Supplementary File S2 and NF-κB, Supplementary File S3) and components of signaling pathways (e.g., TRAF1, Figure 1). Conversely, other genes known to negatively regulate the activation state of the cell were also found among those up-regulated in the host response common to the bacteria and viruses tested. Among these, are: (i) NFκBIA (Supplementary File S2) and NFκBIE (Figure 1) that encode IκB-α and IκB-ε, respectively, and sequester NF-κB in the cytoplasm, blocking its translocation to the nucleus (Hoffmann et al., 2002), (ii) TRAFD1 (Figure 1), which is a negative regulator of Toll-like receptor (TLR) and RIG-I-like signaling pathways (Mashima et al., 2005; Sanada et al., 2008), and (iii) the deubiquitinase TNFα induced protein 3 (TNFAIP3/A20, Figure 1), which is a master regulator of inflammation and the innate immune response (Catrysse et al., 2014). For instance, TNFAIP3/A20 restricts NOD-mediated NF-κB activation by deubiquitination of RIP2 (Hasegawa et al., 2008; Hitotsumatsu et al., 2008) and, in addition, inhibits TLR and RIG-I signaling that activate NF-κB and IRF3/IRF7 transcription factors (Wang et al., 2004; Saitoh et al., 2005; Lin et al., 2006; Shembade et al., 2010). Collectively, it was thus observed that the interaction of pathogens with macrophages induces genes that promote the host innate immune response, needed to control the invading pathogen, but also genes that limit the activated state of the cell, required once infection is resolved.

### Apoptosis

**Figure 2:**
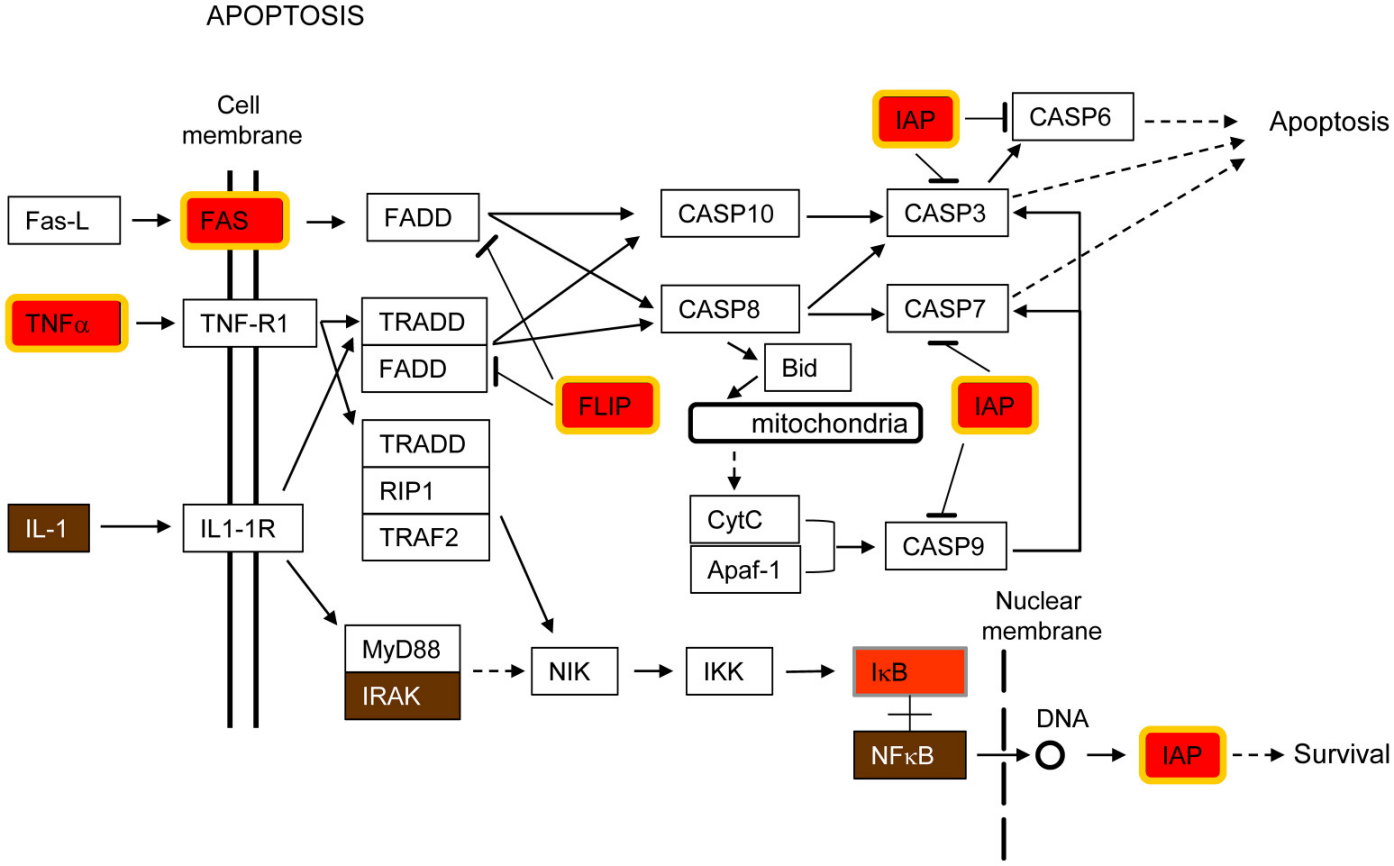
**Apoptosis pathway**. This pathway is a simplified version of the Apoptosis pathway from the Kyoto Encyclopedia of Genes and Genomes (KEGG), shown in Supplementary Figure S1. Only those parts of the KEGG pathway that are relevant for this study are shown in the Figure. Host cell genes commonly up-regulated by *K. pneumoniae, S. pneumoniae, S. aureus* and RSV are highlighted in red and yellow border (FC ≥ 2, FDR ≤ 0.1), red (FC ≥ 2, no FDR), or brown (FC ≥ 1.5, no FDR), depending on the statistical parameters being considered. This color-code is also used in Supplementary Figures S1–S4 and in Figures 3–5.

Apoptosis is a mechanism of induced cell death that is highly regulated by a complex interaction of pro- and anti-apoptotic molecules (Fadeel and Orrenius, 2005). Genes encoding both types of proteins were up-regulated by all pathogens in this study (Tables 2, 3). Supplementary Figure S1 shows a diagram of the KEEG apoptosis pathway highlighting the proteins whose expression was enhanced in infected cells. Figure 2 is a simplified version of the same pathway including those genes up-regulated by the pathogens under study. Thus, Fas cell surface death receptor (Fas), a key molecule in initiating caspase-mediated apoptosis, was up-regulated but, at the same time, baculoviral IAP repeat containing 3 (BIRC3/cIAP2, IAP in Figure 2) and FADD-Like apoptosis regulator (CFLAR/FLIP, FLIP in Figure 2), both having anti-apoptotic functions, were also up-regulated (Figure 2).

The extrinsic or death receptor-mediated pathway of apoptosis is activated by cytokine type receptors for tumor necrosis factor (TNF), TNF-related apoptosis-inducing ligand (TRAIL) and Fas ligand (FasL) (Fadeel and Orrenius, 2005). The binding of ligands to death receptors triggers intracellular signaling that results in the activation of a cascade of caspases within the cell that finally leads to apoptosis (Fadeel and Orrenius, 2005). Figure 2 shows that production of TNFα and Fas receptor was enhanced by all pathogens under study, indicating that the death receptor route to apoptosis is an important host response to these invading pathogens. This pathway is important for the maintenance of tissue homeostasis, especially in the context of immune system function (Danial and Korsmeyer, 2004).

In contrast, anti-apoptotic genes were also up-regulated by all pathogens examined (Figure 2). Thus, CFLAR/FLIP (FLIP in Figure 2), which is an inhibitor of the extrinsic apoptotic signaling by preventing caspase-8 recruitment upon ligation of Fas, was markedly up-regulated (Fadeel and Orrenius, 2005). Additionally, BIRC3/cIAP2 (IAP in Figure 2) which binds TNF-associated factor 2 (TRAF2) in the TNF receptor 1- and 2-associated complexes, where it suppresses caspase-8 activation and apoptosis, was also up-regulated (Wang et al., 1998, 2008). Thus, CFLAR/FLIP and BIRC3/cIAP2 are two examples of anti-apoptotic genes up-regulated in the host response common to all the investigated pathogens.

Both pro- and anti-apoptotic genes were also up-regulated by LPS alone in this study (Figure 1), in consonance with previous reports showing that stimulation of alveolar macrophages with LPS in concert with IFN-γ induces the expression of genes related to apoptosis (Derlindati et al., 2015).

Overall, the results obtained evidence the occurrence of a subtle but complex equilibrium between pro- and anti-apoptotic signals during host-pathogen interaction. Apoptosis is usually beneficial for the host, since apoptotic clearance of infected cells can inhibit microbial replication and propagation (Lamkanfi and Dixit, 2010). In addition, tissue injury is reduced with this type of cell death. While suppression of apoptosis may facilitate replication and proliferation of intracellular pathogens (Clark and Maurelli, 2007), viruses and bacteria can also induce apoptosis to eliminate cells required for a protective immune response (Suzuki et al., 2005). Indeed, in certain infections, apoptosis is first inhibited to facilitate replication of the pathogen and later induced by the host to eliminate the infected cells. Hence, the up-regulated host genes of Figure 2 may represent some of the key players in this arms race between the host and the invading pathogen.

### Pathogen recognition receptors and signaling pathways

Pathogen recognition is the first step in the cascade of cellular events leading to the host defense response. Bacteria and viruses are recognized by different PRRs. However, we found that genes from the three major PRRs pathways (Toll-like, RIG-I-like and NOD-like) were induced by all the pathogens under study (Table 3).

#### Toll-like receptors (TLRs) signaling pathway

TLRs are transmembrane proteins with an extracellular or intra-vesicular domain that mediate recognition of pathogen-associated molecular patterns (PAMPs) and a cytoplasmic domain that initiates downstream signaling (Kumar et al., 2009). To date, 10 TLRs have been identified in humans and 13 in mice. TLRs 1–9 are conserved in both species. According to their cellular localization, TLRs have been classified in two groups: cell surface expressed (TLR1, 2, 4, 5, 6, and 11) and intracellularly expressed (TLR3, 7, 8, and 9). It has been described that the former group senses primarily bacterial wall components, whereas the latter group is involved in the recognition of viral and bacterial nucleic acids (Kumar et al., 2011).

**Figure 3:**
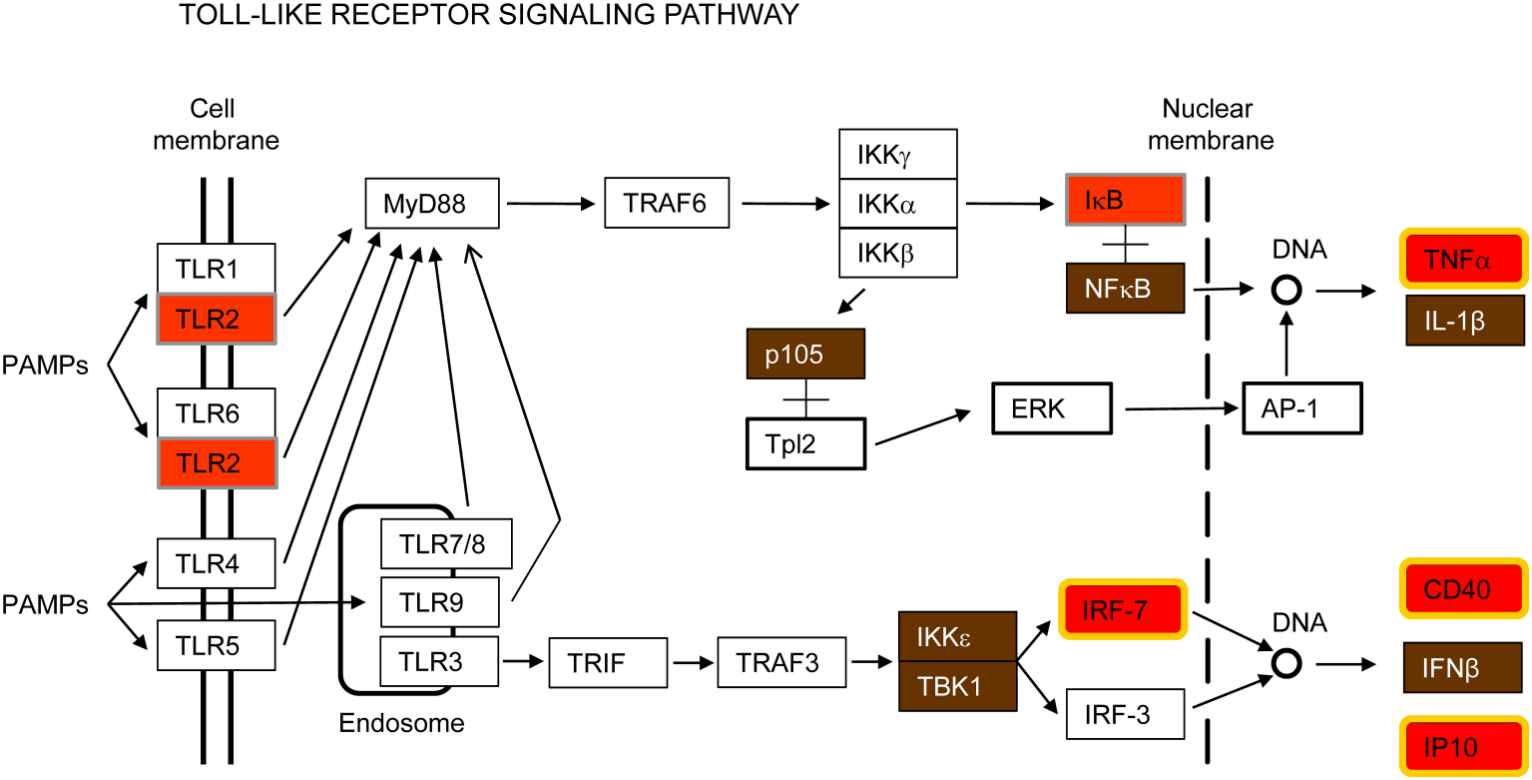
**Toll-like receptor signaling pathway**. This is a simplified version of the KEGG pathway, shown in Supplementary Figure S2.

Toll-like receptor 2 (TLR2) was the only TLR induced by all pathogens tested in this study (Figure 3 and Supplementary Figure S2). TLR2 has been reported to recognize a wide range of PAMPs, including bacterial lipoproteins, peptidoglycans and lipoteichoic acids as well as fungi and viral proteins (Olive, 2012; Lester and Li, 2014). TLR2 is a cell surface receptor expressed in immune cells such as macrophages that signals through the adapter molecule MyD88 to activate NF-κB for transcription of innate immune genes (Figure 3). It has been considered for a long time that activation of TLR2 does not induce interferon (IFN) production. However, it has also been reported that in certain monocytes TLR2 can induce type I IFN in response to viral and bacterial infections through IRF3 and IRF7 activation (Barbalat et al., 2009; Stack et al., 2014). In our study, NF-κB, IRF7 and IFN-β genes were up-regulated by all pathogens tested (Figure 3). Other inflammatory cytokines such as TNFα and CXCL10/IP10 were also up-regulated, as well as the gene encoding the CD40 (cluster of differentiation 40) receptor, necessary for macrophage activation (Figure 3). Thus, TLR2 overexpression in macrophages seems to contribute to recognition of respiratory viruses and bacteria and initiation of the early innate immune response.

#### RIG-I-like receptors (RLRs) signaling pathway

**Figure 4:**
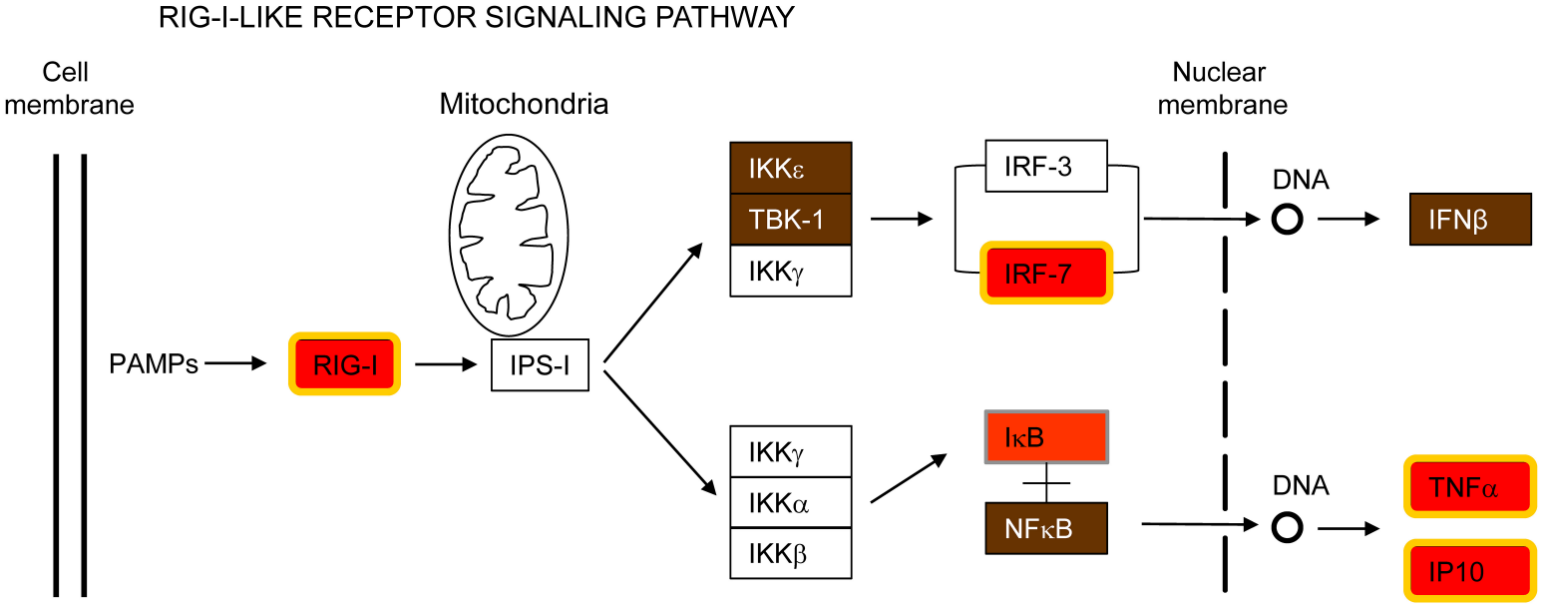
**RIG-I-like receptor signaling pathway**. This is a simplified version of the KEEG pathway, shown in Supplementary Figure S3.

DEAD (Asp-Glu-Ala-Asp) box polypeptide 58 (DDX58 /RIG-I) is also part of the common host response against the respiratory pathogens analyzed in this report (Figure 4 and Supplementary Figure S3). RIG-I is a cytosolic PRR involved in the recognition of pathogen-specific 5′-di or -triphospate RNA (Hornung et al., 2006; Pichlmair et al., 2006; Goubau et al., 2014), leading to activation of IRF7 and subsequent transcription of IFN-β and other immune defense genes (Patel and García-Sastre, 2014). RIG-I is considered to be essential for the innate immune response against many viruses by recognizing blunt-ended base-paired RNAs with a 5′-triphosphate in their genome or in replication intermediates (Kato et al., 2006; Rehwinkel et al., 2010; Weber et al., 2013). However, recent reports have shown that RIG-I is also able to sense bacterial mRNA that translocates to the cytosol (Abdullah et al., 2012; Hagmann et al., 2013; Schmolke et al., 2014). These mRNAs are uncapped and 5′-triphosphorylated. Therefore, the enhanced expression of RIG-I, IRF7, IFN-β, TNFα, and CXCL10/IP10 in MH-S cells infected by all the pathogens examined in the present study extends previous findings and emphasizes a central role of this signaling pathway for triggering the innate immune response against both bacteria and viruses (Figure 4). It is worth noting that RIG-I was not induced in MH-S macrophages exposed to LPS (Figure 1) suggesting that RIG-I induction in this cell type may require intracellular uptake of the invading pathogen.

#### iii) NOD-like receptors (NLRs) signaling pathways

**Figure 5:**
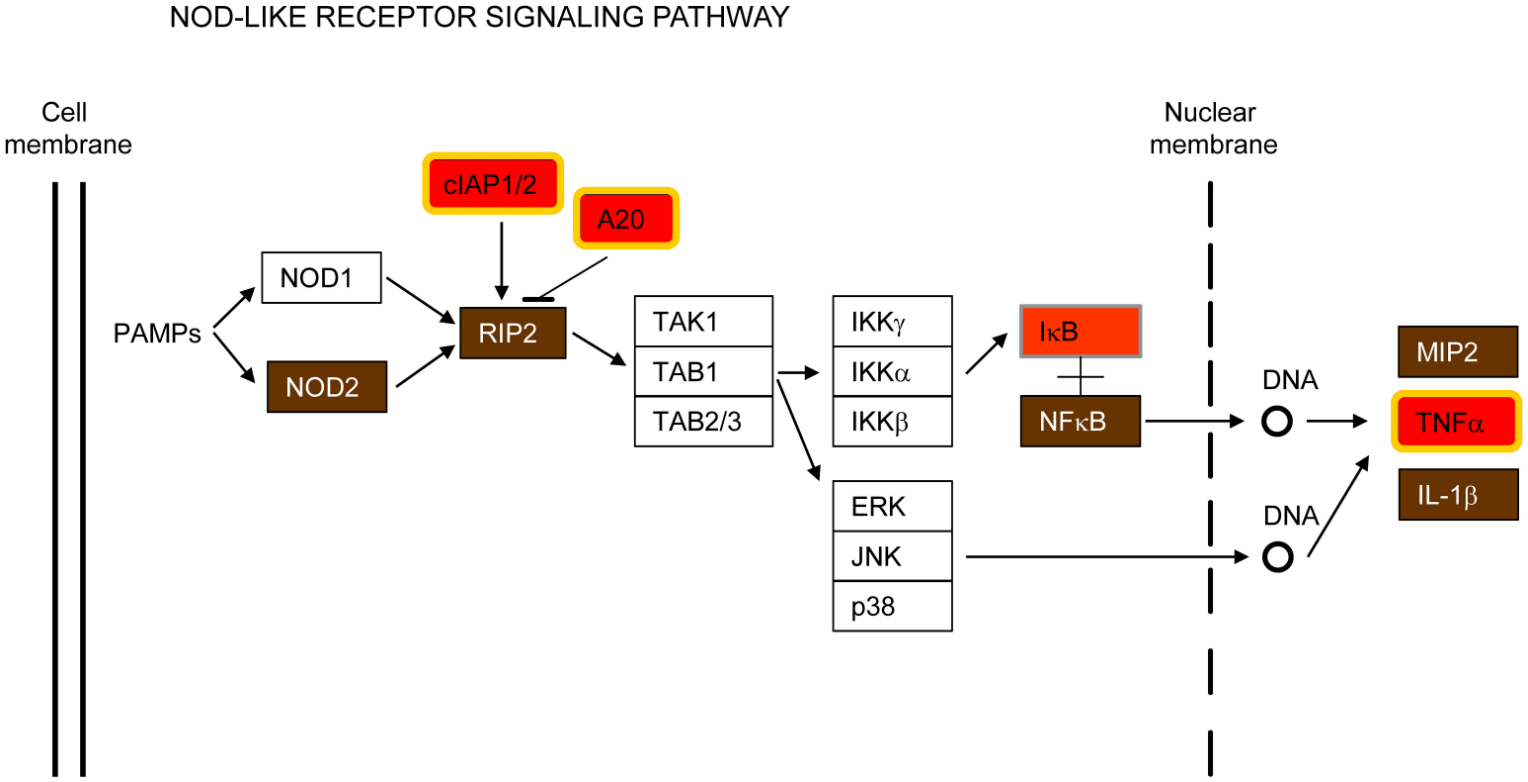
**NOD-like receptor signaling pathway**. This is a simplified version of the KEGG pathway, shown in Supplementary Figure S4.

Nucleotide-binding oligomerization domain-containing 2 (NOD2) is another cytosolic PRR that was also found to be up-regulated in the common host-transcriptional response to all the pathogens examined (Figure 5 and Supplementary Figure S4). NOD2 expression seems to be restricted to macrophages, neutrophils, dendritic and bronchial cells (Qiu et al., 2013; Leissinger et al., 2014), where it recognizes bacterial and viral components triggering the activation of NF-κB signaling that leads to expression of hundreds of genes involved in the innate immune response (Girardin et al., 2003; Inohara et al., 2003). NOD2 has been shown to sense several respiratory bacteria (Opitz et al., 2004; Ferwerda et al., 2005; Kapetanovic et al., 2010; Davis et al., 2011) and to respond to RSV ssRNA, triggering activation of IRF3 (Sabbah et al., 2009). Furthermore, NOD2 interacts with 2′,5′-oligoadenylate synthetase type 2 (OAS2) and activates RNase L, which hydrolyzes viral RNA (Dugan et al., 2009; Ting et al., 2010).

In addition to NOD2, the receptor-interacting serine-threonine kinase 2 (RIPK2/RIP2, RIP2 in Figure 5), a key adaptor molecule of the NOD-like receptor signaling pathway, was also found up-regulated in this study (Figure 5). It is worth noting that RIP2 is also important for the activation of the NF-kB and MAPK pathways, as well as for induction of apoptosis (McCarthy et al., 1998; Girardin et al., 2001; Park et al., 2007; Windheim et al., 2007; Magalhaes et al., 2011).

### Apoptosis and immune signaling pathways are interconnected

Programmed cell death and immune pathways induced by pathogens are closely intertwined and frequently one pathway is replaced by the other in a coordinated battle inside the host for pathogen survival versus elimination (Blander, 2014). In this report, several genes involved in both apoptosis and immune signaling pathways, such as IAP, CFLAR/FLIP and RIPK2/RIP2, were up-regulated by all pathogens tested (Figures 2, 5). As indicated by their name, the inhibitors of apoptosis (IAP) were first described as proteins with anti-apoptotic functions by interfering with the proteolytic activity of caspases (Figure 2). More recently, however, the IAPs have been shown to regulate inflammatory and innate immune signaling pathways through their ubiquitin-ligase activities (Gyrd-Hansen and Meier, 2010; Beug et al., 2012; Estornes and Bertrand, 2015). In addition, CFLAR/FLIP, which inhibits caspase-8 and suppresses apoptosis (Figure 2), has also been shown to activate ERK and increase NF-κB activation in T cells, and to inhibit myeloid cell innate signaling (Kataoka et al., 2000; Wu et al., 2015). Finally, RIPK2/RIP2, which activates NF-κB and MAPKs through binding to NOD2 (Figure 5), has also been shown to induce apoptosis (McCarthy et al., 1998; Thome et al., 1998).

### Future developments

Some of the up-regulated genes identified in this study participate in several of the pathways listed in Table 3 and illustrated in Figures 2–5, indicating a high degree of interconnection and possible redundancy of the host response to viral and bacterial pathogens. Overexpression of some of the gene-products listed in Files S1-S3 have been reported by a number of laboratories using different techniques for quantitation of protein levels. To name a few examples, increased amounts of CXCL10 were demonstrated by Luminex analysis in the supernatant of murine macrophages infected with IAV (Zinman et al., 2011) and increased INF-β levels have been detected by western blots in macrophages infected with RSV (Pokharel et al., 2016) or by ELISA in the supernatant of cells infected with *S. pneumoniae* (Rogers et al., 2003). Therefore, it is likely that the up-regulation of genes listed in Files S1-S3 will be reflected in overexpression of the respective gene products, something that requires further in-depth proteomic analysis. It is worth mentioning, however, that modulation of protein activity may also occur by post-translational modifications that could not be detected by microarray analysis. Hence the gene products highlighted in Figures 2–5 may be only a subset of those implicated in the host response to respiratory pathogens. Nevertheless, the up-regulated genes can be considered as spotlights of the host response and thus first choice for further proteomic and functional studies aimed to unravel the intricacies of the macrophage response to invading respiratory pathogens.

The up-regulated genes identified in this study may also be used in the search of new biological or chemical compounds to fight respiratory infections in at least three different directions:

1. Discovery of new drugs with therapeutic potency against a wide range of respiratory pathogens; as it has been recently documented for IRF3 agonists that have demonstrated antiviral activity against members of the *Flaviviridae* (west Nile virus), *Filoviridae* (Ebola virus), *Orthomyxoviridae* (IAV), *Arenaviridae* (Lassa virus), and *Paramyxoviridae* (RSV) families (Pattabhi et al., 2015).
2. Modulation of the inflammatory response. Some of the most severe pathological symptoms of respiratory infections are commonly caused by inappropriate inflammatory responses. As recently summarized (Estornes and Bertrand, 2015), it has been found that some members of the antiapopototic protein (IAP) family modulate the NOD-like responses to intracellular bacteria and influence the formation of cytoplasmic inflammasomes. In a similar manner, modulation of other up-regulated genes shown in Figures 1–5 might contribute to control the undesirable inflammatory responses associated with respiratory infections. In this respect, it has been shown that up-regulation of TNFAIP3/A20 down-regulates the NF-κB-mediated innate immune response (Catrysse et al., 2014).
3. Vaccine development. TLR ligands have recently found a renovated impulse as vaccine adjuvants (or co-adjuvants) (review by Coffman et al., 2010). Some recently licensed adjuvants (e.g., oil-in-water emulsions, such as MF59 and AS03) that modulate innate immune responses have been shown not only to enhance the protective antibody response but additionally to influence the polarization of this response toward a Th1 or Th2 phenotype or influence mucosal immunity (Gutjahr et al., 2016). Indeed, members of nearly all of the PRR families are potential targets for adjuvants, hence new opportunities are opened to test ligands of the up-regulated gene-products identified in this study as new vaccine adjuvants.

## Conclusions

We have identified more than 30 cellular genes whose expression is significantly enhanced in a macrophage cell line experimentally exposed to different bacteria and viruses of clinical relevance. This approach overcomes possible pitfalls when comparing datasets obtained from studies using seemingly unrelated experimental conditions. While viruses, such as IAV and RSV, are obligatory intracellular parasites, the life cycles of the three bacteria used in this study are mostly extracellular. However, macrophage internalization of *S. pneumoniae* (Gordon et al., 2000), *K. pneumoniae* (Cano et al., 2015), and *S. aureus* (Koziel et al., 2009) has been reported, as well as bacteria survival during several hours/days inside the cell. The experimental conditions used in this work did not allow us to distinguish between the effects triggered by the interactions of the respective pathogens with the target cell before or after internalization, something that merits future investigations.

The up-regulated genes shown in Figure 1 are part of an ostensible core of the cell response to bacterial and viral pathogens which is expanded by other genes included in Supplementary Files S2, S3. There are two essential components in these host-pathogen interactions shared by respiratory viruses and bacteria: (i) recognition of the invading pathogens by collective PRRs and (ii) induction of apoptosis. Both components are intimately integrated to ensure a better control of the infecting microorganisms. The identification of genes up-regulated by different respiratory pathogens reported here should facilitate the search of new host-oriented broad-spectrum medicines.

## Author contributions

Conceived and designed the experiments: IM, VA, CC, EG, JG, MM, DM, AN, JO, CP, VR, JY, JB, and JM. Performed the experiments: IM, Ad, BG, MG, AL, AP, ER, AR, and DS. Analyzed data: IM, JO, IC, and Jd. Wrote the paper: IM, JO, and JM with contributions from all other authors.

### Conflict of interest statement

The authors declare that the research was conducted in the absence of any commercial or financial relationships that could be construed as a potential conflict of interest.

## Footnotes

^1^http://www.unicef.org/publications/files/Pneumonia_The_Forgotten_Killer_of_Children.pdf.

^2^http://www.firsnet.org/images/firs/FIRS-report-for-web.pdf.
