# Supplementary figures and images for: Apoptosis, Toll-like, RIG-I-like and NOD-like Receptors Are Pathways Jointly Induced by Diverse Respiratory Bacterial and Viral Pathogens
Source: Front Microbiol. 2017 Mar 1;8:276. doi: 10.3389/fmicb.2017.00276 (PMC5331050; doi:10.3389/fmicb.2017.00276)

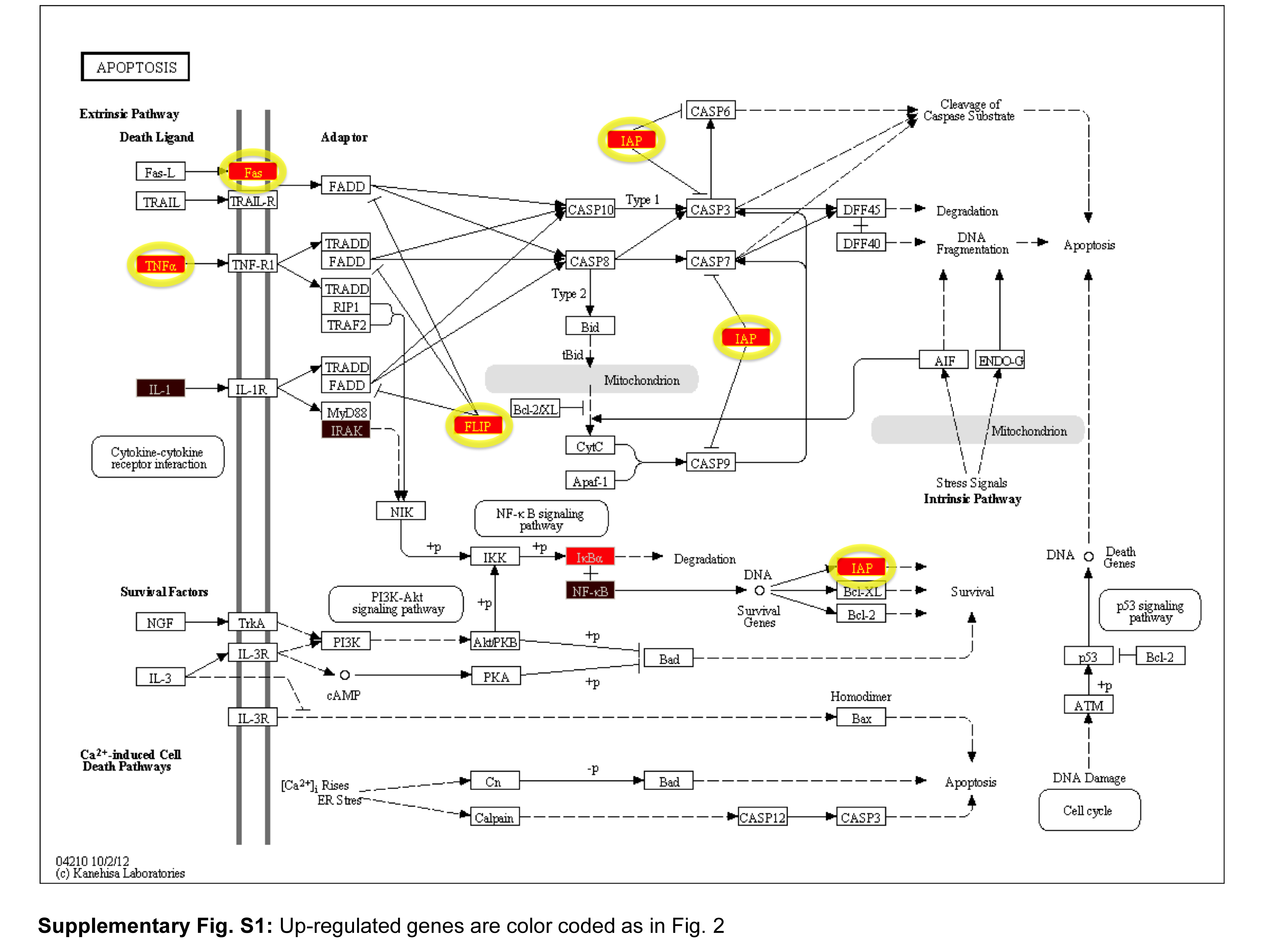

Supplement: Supplementary file 4 [file Image1.TIFF]

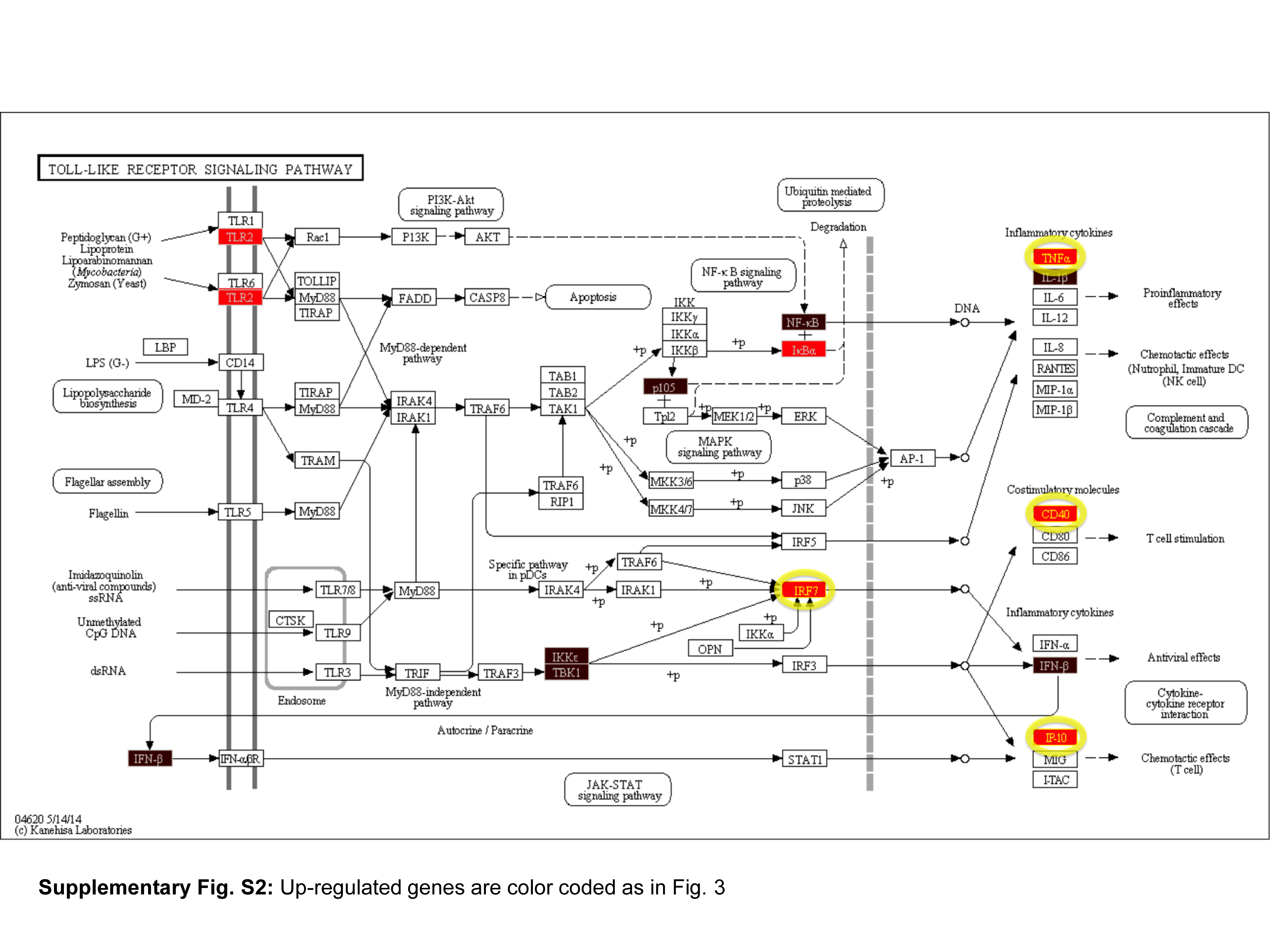

Supplement: Supplementary file 5 [file Image2.TIFF]

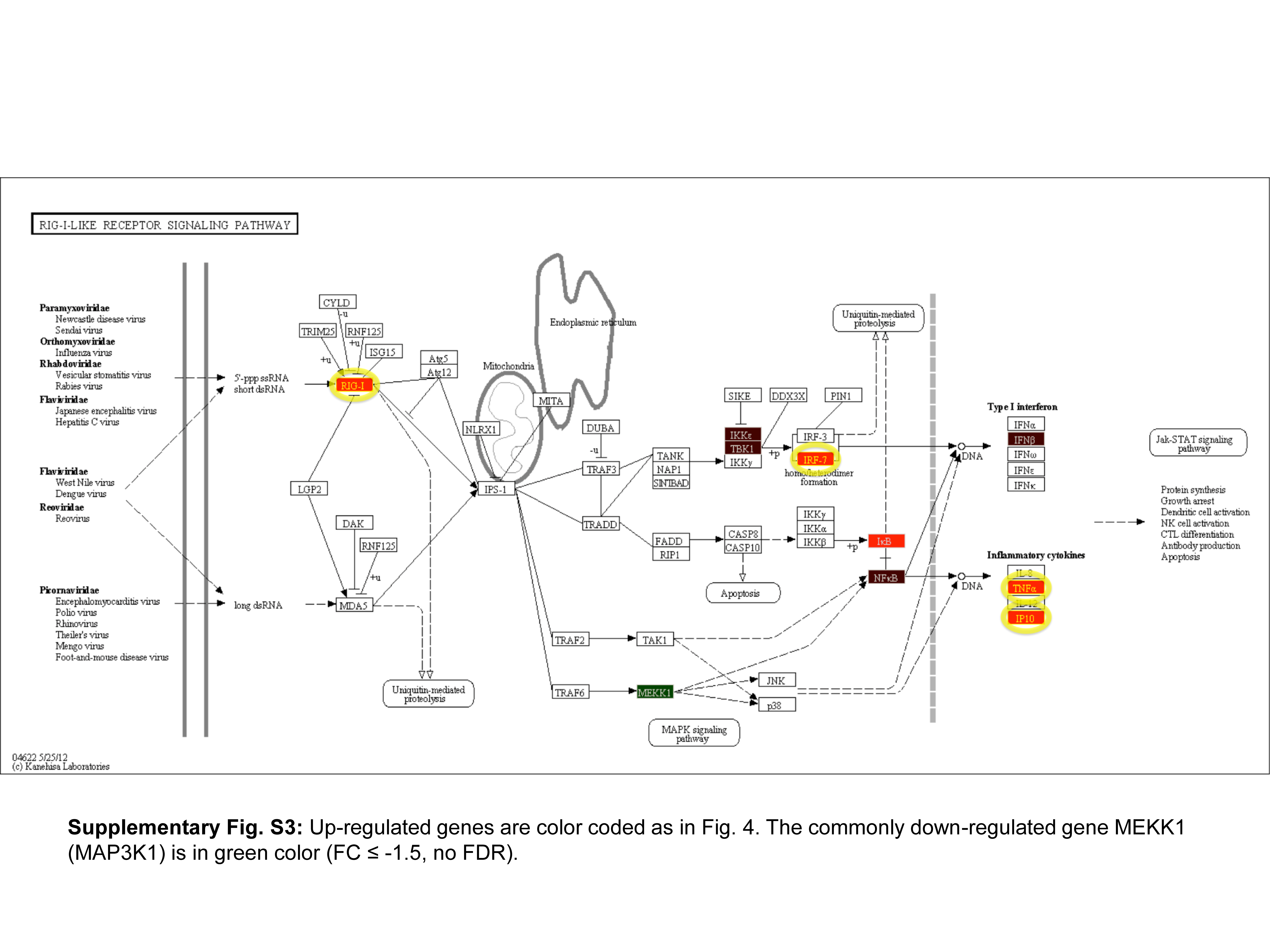

Supplement: Supplementary file 6 [file Image3.TIFF]

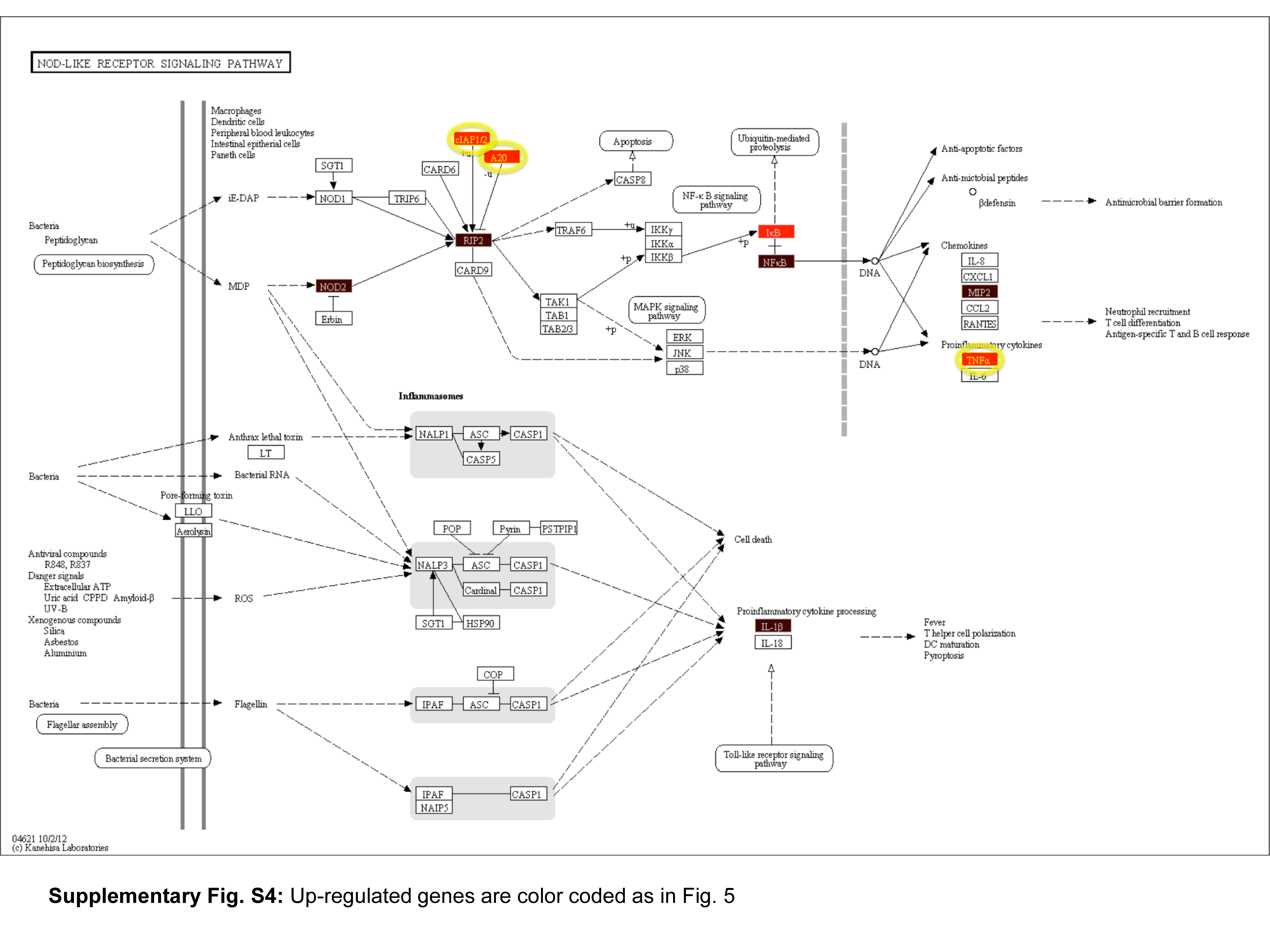

Supplement: Supplementary file 7 [file Image4.TIFF]
